# Overlapping structural and functional connectivity disruptions in clinical high-risk for psychosis participants: A network analysis study

**DOI:** 10.1016/j.nicl.2025.103803

**Published:** 2025-05-12

**Authors:** Angus Paton, Tineke Grent- ’t-Jong, Ruchika Gajwani, Joachim Gross, Andrew I. Gumley, Stephen M. Lawrie, Matthias Schwannauer, Frauke Schultze-Lutter, Peter J. Uhlhaas

**Affiliations:** a Institute for Neuroscience and Psychology, Univ. of Glasgow, UK; b Department of Child and Adolescent Psychiatry, Charité Universitätsmedizin, Berlin, Germany; c Institute of Health and Wellbeing, Univ. of Glasgow, UK; d Institute of Biomagnetism and Biosignalanalysis, University of Muenster, Muenster, Germany; e Department of Psychiatry, Univ. of Edinburgh, UK; f Department of Clinical Psychology, Univ. Edinburgh, UK; g Department of Psychiatry and Psychotherapy, Medical Faculty, Heinrich Heine University and University Clinic, Düsseldorf, Germany; h University Hospital of Child and Adolescent Psychiatry and Psychotherapy, University of Bern, Bern, Switzerland; i Department of Psychology, Faculty of Psychology, Airlangga University, Airlangga, Indonesia

**Keywords:** CHR-P, Diffusion MRI, resting state fMRI, Connectome, Correlation

## Abstract

Schizophrenia involves aberrant connectivity of anatomical and functional networks. However, it is currently unclear whether such disruptions are present in clinical high-risk for psychosis (CHR-P) participants and whether there may be an overlap between structural and functional connectivity alterations.

We obtained diffusion magnetic resonance imaging (dMRI) and resting-state, magnetic resonance imaging (rsfMRI) data from N = 110 CHR-P participants and N = 49 healthy controls (HC). A network analysis approach was employed to explore differences in dMRI and rsfMRI connectivity as well as potential overlap between both modalities. In addition, correlations between dMRI and rsfMRI-data, clinical and neurocognitive variables as well as with clinical outcomes were investigated.

We observed hyper- and hypoconnectivity across occipital, parietal, temporal, and frontal cortices in both dMRI and rsfMRI-data in CHR-Ps. Moreover, dMRI- and rsfMRI-defined overlapping nodes that differed between CHR-Ps vs. HC overlapped with visual networks, right ventral attention network, and right default mode network. Correlational analyses indicated significant relationships between the severity of CHR-P symptoms, cognitive deficits, and dMRI/rsfMRI-defined hypo- and hyper-connectivity. Finally, CHR-P individuals with persistent attenuated psychotic symptoms (APS) were characterised by aberrant dMRI and rsfMRI connectivity compared to non-persistent APS.

Our study shows subtle but widespread disruptions in dMRI and rsfMRI connectivity in CHR-P participants. Specifically, we identified several occipital, parietal, temporal, and frontal regions that were characterised by hyper and hypoconnectivity which correlated with the severity of clinical symptoms and cognitive impairments. Moreover, our findings suggest that dMRI and rsfMRI connectivity measures could serve as a potential biomarker for clinical outcomes in CHR-Ps.

## Highlights

• Hyper- and hypoconnectivity connectivity disruptions were found in CHR-P participants in occipital, parietal, temporal, and frontal regions.
• Overlapping alterations in DTI and rsfMRI data were identified in auditory, cingulo-opercular, frontoparietal and visual networks.
• Connectivity measures were associated with clinical symptoms cognitive deficits, and functional impairments in CHR-P participants.
• CHR-P individuals with persistent attenuated psychotic symptoms (APS) showed distinct DTI and rsfMRI connectivity patterns.
• DTI and rsfMRI connectivity measures could serve as biomarkers for early detection and prediction in CHR-P individuals.

## Graphical abstract

**Figure:**
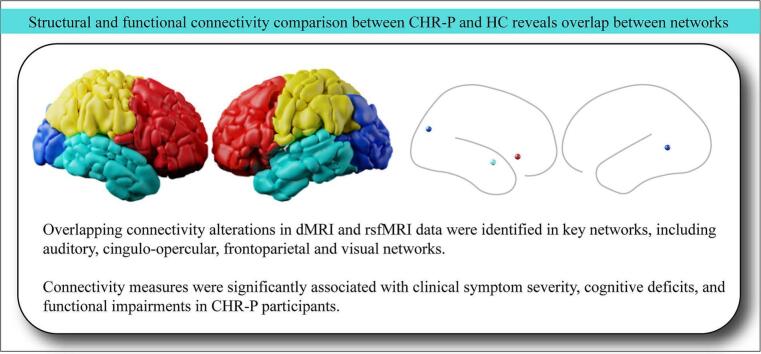

## 1 Introduction

Converging evidence suggests that schizophrenia involves alterations in the structural and functional organisation of brain networks (Friston et al., 2016, Fornito et al., 2012). Since the early formulation of the clinical concept, the notion of a “dysconnectivity syndrome” has highlighted that disturbances in anatomical connections between and within brain regions underlie clinical symptoms and cognitive deficits (Wernicke, 1906). This hypothesis has received extensive support through anatomical imaging studies in schizophrenia that identified extensive cortical and subcortical abnormalities in white and grey matter (WM/GM) (Friston et al., 2016, Sapienza et al., 2022, Harikumar et al., 2023).

More recently, network analysis methods derived from graph theory, such as the Network Based Statistic (Zalesky et al., 2010), have been applied towards the analysis of structural and functional magnetic resonance imaging (MRI/fMRI) data in schizophrenia (Zalesky et al., 2010, Nelson et al., 2017)**.** An important property of brain networks is a structural connectome characterised by a central rich club core (van den Heuvel and Sporns, 2011). These are a group of highly connected, key brain regions that are centrally located, densely interconnected with each other, and serve as central hubs within the brain's overall connectivity network. Disruptions in their connectivity may be linked to the symptoms and cognitive deficits observed in individuals with schizophrenia (Fornito et al., 2012, van den Heuvel and Fornito, 2014, Wannan et al., 2019) and those at genetic high-risk (Collin et al., 2017).

More recently, graph-theoretical methods have been developed to investigate the overlap between structural and functional differences in patients with schizophrenia (Zalesky et al., 2010, Nelson et al., 2017, McNally, 2021). Studies using network methods identified converging structural and functional changes in distributed networks involving cortical, sub-cortical and cerebellar regions in schizophrenia (Zalesky et al., 2010, Nelson et al., 2017, Liu et al., 2021, Gifford et al., 2020, McNally, 2021). However, it is currently unclear whether such overlapping deficits between structure and functional connectivity are also present in individuals at clinical high-risk for developing psychosis (CHR-P).

Schizophrenia is preceded in most cases by a CHR-P state lasting approximately 5–6  years (Fusar-Poli et al., 2013). Diagnostic categories include attenuated psychotic symptoms (APS), a genetic risk and functional deterioration (GRFD) syndrome, and brief limited intermittent psychotic symptoms (BLIPS) (Fusar-Poli et al., 2013, Schultze-Lutter et al., 2012). Moreover, CHR-P criteria have been established based on the presence of basic symptoms (BS) (Schultze-Lutter et al., 2012), which describe subjectively experienced disturbances in speech, attention, and perception. Approximately 25 % of CHR-P participants will develop a first-episode of psychosis (FEP) within a 3-year period (Fusar-Poli et al., 2020). However, transition risk varies significantly across studies and is impacted by recruitment pathways (Fusar-Poli et al., 2015) and clinical characteristics of CHR-P participants (Fusar-Poli et al., 2018).

There is emerging evidence that CHR-P participants are characterised by alterations in both large-scale functional (Collin et al., 2018, Bernard et al., 2017, Vargas, 2019) and structural connectivity (Bernard et al., 2017), suggesting the possibility that these parameters could lead to insights into pathophysiological mechanisms and biomarkers for early detection and prognosis (Yung and McGorry, 1996). Dysconnectivity in large-scale networks may also underlie cognitive deficits as well as clinical symptoms as shown by associations between alterations in connectivity patterns and cognitive deficits (Cao et al., 2018) and prodromal symptoms (Anticevic et al., 2015) in CHR-P participants.

CHR-P participants are characterised by anatomical deficits as indicated by reductions in GM in frontal, temporal, and cingulate cortical regions (Merritt et al., 2021, Jalbrzikowski et al., 2021), while WM abnormalities have been observed in the superior longitudinal fasciculus, cingulate gyrus WM, right anterior corona radiata, right fornix stria terminalis, and left tapetum (Kristensen et al., 2019, Biase et al., 2021). Diffusion MRI studies have revealed reduced fractional anisotropy and increased radial diffusivity in CHR-P who convert to psychosis (Waszczuk et al., 2021), particularly in the splenium (Smigielski et al., 2022). DMRI-deficits in CHR-Ps also correlate significantly with positive symptoms (Nägele et al., 2020). Moreover, resting-state fMRI studies have identified widespread disruption in a cerebro-thalamo-cortical network (Bernard et al., 2017, Anticevic et al., 2015)which predict clinical outcomes, such as transition to psychosis (Anticevic et al., 2015). However, how aberrant anatomical connectivity impacts functional brain networks in CHR-Ps remains unclear.

In the current study, we aimed to provide further evidence for the disruption of functional and anatomical network organisation in CHR-P participants and their potential overlap through investigating dMRI as well as rsfMRI data in N = 110 CHR-P participants and N = 49 healthy controls with a Network Based Statistic (Zalesky et al., 2010) approach. Moreover, we explored relationships with clinical symptoms, functioning and cognitive impairments.

## 2 Methods

### 2.1 Participants

This study recruited a total of N = 159 participants from the Youth Mental Health Risk and Resilience (YouR) Study (Uhlhaas et al., 2017), including N = 110 participants meeting CHR-P criteria and 49 healthy control participants (HC) without an axis I diagnosis or family history of psychosis. CHR-P status was established using the ultra-high-risk criteria according to the Comprehensive Assessment of At-Risk Mental States (CAARMS) Interview (Yung et al., 2005) and the Cognitive Disturbances (COGDIS) and Cognitive-Perceptive (COPER) basic symptoms criteria as established by the Schizophrenia Proneness Instrument–Adult (SPI-A) version interview (Schultze-Lutter et al., 2012). Cognition was assessed with the Brief Assessment of Cognition in Schizophrenia (BACS) (Keefe et al., 2004) battery.

The study was approved by the NHS (National Health Service) Research Ethical Committee Glasgow & Greater Clyde. All participants gave written informed consent.

#### 2.1.1 Participant Follow-Up

We assessed participants meeting CHR-P criteria at 3-, 6-, 9-, 12-, 18-, 24-, 30-, and 36-month intervals using the CAARMS interview to examine the persistence of APS and functional outcomes (Table 1). For the follow-up analyses, we used Global Assessment of Functioning (GAF) data from 6- and 12-months follow-ups. We operationalised the persistence of ultra-high-risk criteria by the continued presence of APS up to 12 months.

**Table 1:** Demographic, clinical, functional and cognitive characteristics of CHR-P (N = 123) and HC (N = 49) participants at baseline.

	**CHR-P** **(N = 123)**	**HC** **(N = 49)**	***p***	**Effect size**^a^
Demographic, clinical and functional data				
Age (years), mean (SD)	21.65 (4.45)	22.63 (3.62)	0.031	*r* = 0.164
Sex, female n (%)	87 (70.7)	33 (67.3)	0.663	*ϕ = 0*.033
Education (years), mean (SD)	15.16 (3.22)	16.61 (3.03)	0.004	*r* = 0.220
Current medication, n (%)				
Antidepressant use	34 (27.6)	0 (0)	< 0.001	*ϕ = 0*.313
Anxiolytic use	12 (9.8)	0 (0)	0.023	*ϕ* = 0.173
Antipsychotic use	3 (2.4)	0 (0)	0.270	*ϕ = 0*.084
Current MINI comorbidity, n (%)				
Anxiety disorder	91 (74.0)	0 (0)	< 0.001	*ϕ* = 0.669
Mood disorder	65 (52.8)	0 (0)	< 0.001	*ϕ = 0*.492
Alcohol abuse/dependence	34 (27.6)	2 (4.1)	< 0.001	*ϕ = 0*.261
Drug abuse/dependence	21 (17.1)	0 (0)	0.002	*ϕ = 0*.235
Eating disorder	9 (7.3)	0 (0)	0.0.52	*ϕ* = 0.148
Current psychological therapy, n (%)	23 (18.7)	0 (0)	0.001	*ϕ* = 0.248
CAARMS severity, median (range)	28 (0–74)	0 (0–12)	< 0.001	*r* = 0.744
SPI-A severity, median (range)	7 (0–74)	0 (0–2)	< 0.001	*r* = 0.707
GAF, median (range)	58 (21–91)	88 (68–97)	< 0.001	*r* = 0.730
GF: Social current, median (range)	8 (3–10)	9 (8–10)	< 0.001	*r* = 0.581
GF: Role current, median (range)	8 (3–9)	9 (5–9)	< 0.001	*r* = 0.519
Mean FD (X, Y, Z (mm))(X, Y, Z (degrees) )Mean Relative RMS (mm)	0.03, 0.13, 0.05, 0.06, 0.02, 0.02 0.22	0.02, 0.1, 0.04, 0.05, 0.01, 0.01 0.18		
Cognitive data, mean (SD)				
Verbal memory	−0.24 (1.23)	0.06 (1.01)	0.102	*d* = 0.256
Working memory	−0.16 (1.46)	0.01 (1.02)	0.756	*r* = 0.024
Motor speed	−0.98 (1.29)	0 (1.00)	< 0.001	*d* = 0.808
Verbal fluency	−0.17 (0.95)	−0.01 (0.99)	0.549	*r* = 0.046
Attention and processing speed	−0.56 (1.12)	0.02 (1.00)	0.001	*d* = 0.533
Executive function	−0.18 (1.32)	−0.01 (0.98)	0.725	*r* = 0.027
BACS total	−0.87 (1.82)	0.02 (0.99)	< 0.001	*d* = 0.548

BACS, Brief Assessment of Cognition in Schizophrenia; CAARMS, Comprehensive Assessment of At-Risk Mental States; CHR-P, clinical high-risk for psychosis; GAF, Global Assessment of Functioning; GF, Global Functioning; HC, healthy control; MINI, Mini-International Neuropsychiatric Interview; SPI-A, Schizophrenia Proneness Instrument, Adult version

a Effect sizes were Rosenthal's *r* for Mann-Whitney U tests (small effect = 0.1, medium effect = 0.3, large effect = 0.5), Cohen’s *d* for Welch’s *t*-tests (small effect = 0.2, medium effect = 0.5, large effect = 0.8) and Phi (*ϕ*) for Pearson’s chi-squared or Fisher′s exact tests (small effect = 0.1, medium effect = 0.3, large effect = 0.5).

### 2.2 Magnetic resonance imaging data Acquisition

We collected dMRI and rsfMRI data at the Centre for Cognitive Neuroimaging, University of Glasgow, using a Siemens 3 T MR Tim Trio system. We obtained a high-resolution T1-weighted anatomical scan for each subject (repetition time = 2300 ms, volumes = 192, voxel size = 1 x 1 x 1 mm). We collected dMRI data (repetition time = 12900 ms, echo time = 100 ms, volumes = 66, voxel size = 1.71875 x 1.71875 x 1.7 mm, b0 value = 1000, diffusion-weighted directions = 59, total scan time = 14 min, 24 s) and resting state imaging data (repetition time = 2000 ms, echo time = 30 ms, volumes = 150, slices = 32, voxel size = 3 x 3 x 3.72 mm, field of view = 210, total scan time = 5 min) in the same session, and also collected field mapping data (repetition time = 488 ms, echo time = 4.92 ms (magnitude), 7.38 ms (phase), slices = 32, voxel size = 3 x 3 x 3.72 mm, field of view = 210).

### 2.3 Image Pre-Processing

We first corrected dMRI-data for spatial distortions with SPM12 using field mapping data collected during the same session. We further corrected the diffusion data for eddy currents (Andersson and Sotiropoulos, 2016) and motion using Oxford Centre for Functional Magnetic Resonance Imaging of the Brain (FMRIB) Software Library in FSL, specifically the FMRIB Diffusion Toolbox (FDT). We then created a brain mask of the non-diffusion weighted image along with text files containing the b-values and gradient orientations for each volume. We ran FSL’s ‘bedpostx’ and ‘probtrackx’ (Behrens et al., 2003, Behrens et al., 2007) to estimate the fibre orientations in each voxel and probabilistic tractography between pairs of seed regions taken from the Gordon2014 atlas.

We pre-processed the resting-state data using CONN toolbox for Matlab (Whitfield-Gabrieli, 2012). This pre-processing involved slice-time scan correction, 3D motion correction, transformation to MNI standard space, segmentation and smoothing with an 8 mm gaussian kernel. We then denoised the data by linear regression using several regressors for noise components that included 5 principal components each for white matter and cerebral spinal fluid, 12 components for subject-motion parameters (current and lagged first order displacement), a variable number of noise components that are identified as outlier volumes (scrubbing), and 2 components that model the effect of rest with the intention to reduce the influence of slow trends. After regression, we then band-pass filtered the data to remove frequencies below 0.008 Hz and above 0.09 Hz.

### 2.4 Constructing diffusion and rsMRI networks

**Fig. 1:**
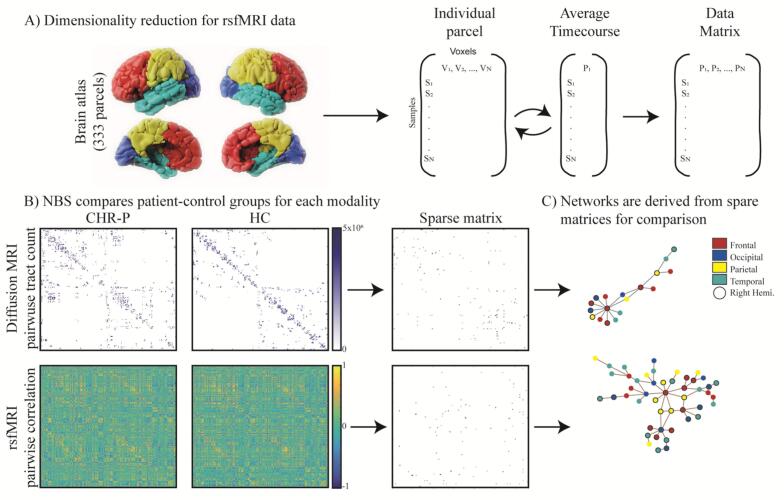
Construction and comparison of dMRI and rsfMRI connectomes.

To investigate differences between dMRI and rsfMRI respectively between CHR-P and HC groups, we constructed pairwise tract count and pairwise Pearson correlation connectomes for each participant (Fig. 1). We selected the ‘Gordon2014′ atlas (Gordon et al., 2014) to use for parcellation of the rsfMRI data by average time course, and to use as seed regions for calculating pairwise tractography. This atlas represents 333 functionally unique brain regions (55,278 unique node pairs) in MNI standard space. The atlas is derived from a rsfMRI data analysis; therefore, it serves as a strong basis for comparing rsfMRI and dMRI in our patient-control study. The atlas has several advantages over alternative parcellations: 1) Each parcel is homogenous, containing a unique resting state functional connectivity signal 2) the parcellation overlaps with known human cortical areas that have been well described with cytoarchitectonics and 3) has a large-scale network structure that is consistent with the experimental data known network structure of the brain. We also chose the Gordon2014 atlas over alternative atlases with fewer parcels because using an atlas with fewer parcels typically results in each parcel combining multiple smaller regions from the Gordon2014 atlas. This merging increases the likelihood of self-loops (Zalesky et al., 2010), where a region is overly connected to itself. The Gordon2014 atlas strikes a balance by providing a finer parcellation that not only avoids issues like self-loops but also offers a more accurate representation of the underlying resting-state functional connectivity.

For the dMRI connectome, we used FDT’s probabilistic tracking to measure connectivity between the 333 regions of interest in the ‘Gordon2014′ atlas with default settings of 5000 samples and a curvature threshold of 0.2 (Behrens et al., 2003, Behrens et al., 2007). As the atlas is in standard MNI space, we used FSL to compute linear and non-linear transforms between each participant’s anatomical space and MNI standard space, and anatomy and diffusion space using the diffusion reference image. Inverse transforms could then be calculated for converting the atlas to each subject space for probabilistic tractography. For rsfMRI, the data was coregistered to MNI template space directly.

We used the Harvard-Oxford cortical atlas to identify brain region labels within each atlas parcel (Makris et al., 2006, Frazier et al., 2005, Desikan et al., 2006, Goldstein et al., 2007) as the Gordon2014 atlas parcel labelling is descriptively sparse, only containing 12 unique labels related to brain networks, not brain regions (Supplementary D). In the Gordon2014 atlas labelling, 42 of the 333 parcels are labelled “None” as they are not related to any of the 12 common network labels. This is a limitation of the Grodon2014 atlas which we remedied by substituting the labels with those from the Harvard-Oxford atlas, increasing the number of labels to 97 (Supplementary E). We refer to the Gordon2014 labels when we consider which parcels belong to which common brain network.

#### 2.4.1 Quality control of dMRI and rsfMRI data and connectomes

Quality control is a critical step for ensuring validity, reproducibility and interpretability in brain network analysis. After pre-processing, data are visually inspected. To ensure quality control of the resulting connectomes, we apply absolute thresholds (Garrison et al., 2015) to remove spurious connectivity (Sotiropoulos, 2017) from the subsequent analysis. DMRI is typically thresholded to around 5–20 counts (De Brito Robalo et al., 2022) and for rsfMRI connectomes thresholding ultimately is a balance between the research question and the type of thresholding approach (van den Heuvel et al., 2017) aimed at eliminating systematic sparsity differences as the primary cause of group differences in patient-control studies. One of the advantages of the Network Based Statistic method adapted here is that it uses the raw measure of connectivity rather than a thresholded binary adjacency matrix, thus reducing the problem of systematic sparsity differences (Zalesky et al., 2010).

Therefore, to remove spurious connectivity, dMRI connectomes were thresholded at 10 counts and rsfMRI connectomes were thresholded at an absolute value of r > 0.05. The rsfMRI connectomes were further normalised using Fisher’s r to z normalisation. The choice of thresholds was a careful consideration to remove spurious connectivity without inducing instability to the underlying networks (Garrison et al., 2015).

To further ensure the quality of our data, we excluded participants from the analysis by comparing their motion parameters (Supplementary A, B, C) to the group average motion. Participants were excluded if they were beyond 1 standard deviation of the group average. For the dMRI analysis, we removed six participant from the HC and seven participants from the CHR-P group. For the rsfMRI analysis, we removed seven HC participants and nine CHR-Ps.

### 2.5 Using Network-Based Statistic Method to compare CHR-P vs. HC groups

To investigate differences between dMRI and rsfMRI connectomes, we implemented a version of the Network Based Statistic method (Zalesky et al., 2010). We compared the vector of values for dMRI (or rsfMRI) between the CHR-P group and controls for each edge weight in the connectome using a Mann Whitney-Wilcoxon non-parametric test with a p-value threshold of p < 0.001. This procedure created a sparse matrix identifying uncorrected significant differences between the connectomes of the two groups that pass the conservative p threshold of 0.001. We determined corrected significant differences using non-parametric permutation testing (10,000 iterations) with random group assignment while maintaining original group sizes. Rather than using a network statistic to reduce computational load, we instead computed permutation testing for each edge weight. We used the resulting null-distribution for each edge to perform a significance test of the observed statistic for the true group comparison with the null-distribution.

To examine the relationship between dMRI and rsfMRI (Nelson et al., 2017) connectivity, we computed the intersect of the largest subnetwork from their respective sparse matrices. We determined the significance of the identities of the nodes that are part of the intersect using non-parametric permutation testing (10,000 iterations) with random group assignment maintaining original group sizes. For each iteration, we recorded the identity of any nodes that were part of the intersect of the dMRI and rsfMRI connectomes. The null distribution of node identity allowed us to perform a significance test for each node identity − of the true group comparison − by dividing the number of times each node identity appeared in the null distribution by the total number of permutations. The resulting p-values were then corrected using the Benjamini and Hochberg false discovery rate method (Benjamini and Hochberg, 1995).

### 2.6 Calculating effect sizes of significant differences between CHR-P vs. HC Groups, and Determining hub nodes

To calculate the effect size and directionality of group differences of the Mann Whitney-Wilcoxon test, we use the following formula:

R=Z/sprt (N)where Z is the z-score obtained from the Mann Whitney test and N is the total number of subjects.

To determine hub nodes within each subnetwork, we computed the eigenvector centrality of each node. This measure considers the degree of the node and its importance in relation to its connectedness with other important nodes (Fornito, 2016). We define hub nodes as having an eigenvector centrality greater than one standard deviation from the mean of the distribution of centrality values for all nodes within the subnetwork (Gong et al., 2008).

### 2.7 Calculating correlations between brain imaging differences and clinical measures

We calculated Pearson correlations between clinical scores (total CAARMS severity, total SPI-A severity, total BACS and GAF scores) with dMRI (or rsfMRI) connectivity node pairs that included one of the intersecting node identities. We controlled the false discovery rate using non-parametric permutation testing (1,000 iterations). We further corrected the resulting p-values using the Benjamini and Hochberg false discovery rate method (Benjamini and Hochberg, 1995).

## 3 Results

### 3.1 Demographic and clinical data

The CHR-P group was slightly younger than the HC group (p = 0.05) and had fewer years of education (p = 0.05) (Table 1). Moreover, CHR-P participants had significantly lower BACS composite (p = 0.001), token motor (p < 0.001), and symbol coding (p = 0.001) scores than the HC group. Finally, the CHR-P group had significantly lower GAF-scores as well as global role and social functioning than HC (p < 0.001).

### 3.2 Diffusion MRI network Analyses: CHR-P vs. HC

Out of 55,278 node pairs, 59 showed significant differences between groups (0.0011 %) of which 52 showed higher connectivity in the HC group and 7 showed higher connectivity in the CHR-P group. Effect sizes ranged from 0.26 to 0.51 with a median of 0.29 (Supplementary K).

**Fig. 2:**
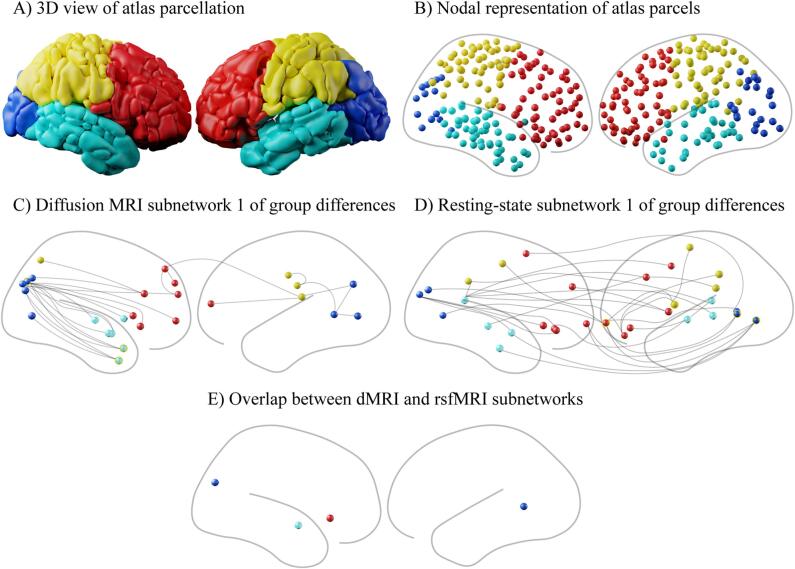
DMRI and rsfMRI subnetworks derived from comparing CHR-Ps and HC.

We identified two subnetworks spanning frontal, occipital, parietal, and temporal regions across hemispheres that differed between CHR-P and HC (Fig. 2C, Table 2, Supplementary F). The largest subnetwork contained three hub nodes, including right cuneal cortex (degree = 8, centrality = 0.61), right temporal pole (degree = 3, centrality = 0.35) and right anterior inferior temporal gyrus (degree = 3, centrality = 0.29). The smaller subnetwork (Supplementary F) contained a single hub node posterior supramarginal gyrus (degree = 5, centrality = 0.66). These data show that dMRI connectivity differences between CHR-P and HC groups are spatially distributed, spanning brain areas involved in visual processing, attention, language, sensory integration, and executive function.

**Table 2:** Node ID and Brain Region for dMRI subnetwork 1 for differences between CHR-P and HC.

**Node ID**	**Region**
12	LH, Cingulate Gyrus, posterior, anterior
15	LH, Intracalcarine Cortex
16	LH, Lingual Gyrus
26	LH, Cingulate Gyrus, posterior
65	LH, Parietal Operculum Cortex
90	LH, Cuneal Cortex
149	LH, Frontal Pole
166	RH, Occipital Pole, Lateral Occipital Cortex, superior
234	RH, Insular Cortex
235	RH, Insular Cortex
242	RH, Frontal Orbital Cortex
248	RH, Insular Cortex
252	RH, Lateral Occipital Cortex, superior
255	RH, Cuneal Cortex, Lateral Occipital Cortex, superior
256	RH, Cuneal Cortex
258	RH, Lateral Occipital Cortex
264	RH, Lateral Occipital Cortex, inferior
273	RH, Middle Frontal Gyrus, Inferior Frontal Gyrus
277	RH, Frontal Pole
292	RH, Temporal Pole
300	RH, Inferior Temporal Gyrus, anterior, Temporal Pole
321	RH, Frontal Pole
323	RH, Frontal Pole
324	RH, Superior Frontal Gyrus
330	RH, Heschl’s Gyrus, Planum Polare, Temporal Pole
331	RH, Superior Temporal Gyrus, anterior

### 3.3 rsfMRI network analyses: CHR-P vs. HC

Out of the 55,278 node pairs, 96 node pairs showed significant differences between groups (0.0018 %), 31 showed higher connectivity in the HC group and 65 in the CHR-P group. Effect sizes ranged from 0.27 to 0.36 with a median of 0.31 (Supplementary L).

We identified three large subnetworks that differ between CHR-Ps and HC groups (Fig. 2D, Table 3, Supplementary H, I). Subnetworks were distributed across bilateral frontal, parietal, temporal and occipital areas. The largest subnetwork (Fig. 2D, Table 3) contained four hub nodes; left frontal pole (degree = 6, centrality = 0.58), left lingual gyrus A (degree = 4, centrality = 0.41), left lingual gyrus B (degree = 2, centrality = 0.3), and left central opercular cortex (degree = 3, centrality = 0.29). The second subnetwork (Supplementary H) contained one hub node left frontal orbital cortex (degree = 6, centrality = 0.66). The third subnetwork (Supplementary I) contained two hub nodes left middle frontal gyrus (degree = 4, centrality = 0.52) and right anterior parahippocampal gyrus (degree = 3, centrality = 0.46).

**Table 3:** Node ID and Brain Region for rsfMRI subnetwork 1 for differences between CHR-P and HC.

**Node ID**	**Region**
64	LH, Supramarginal Gyrus, posterior
69	LH, Parietal Operculum Cortex, Planum Temporale
101	LH, Central Opercular Cortex
108	LH, Middle Frontal Gyrus
111	LH, Central Opercular Cortex
115	LH, Frontal Pole
119	LH, Frontal Orbital Cortex
122	LH, Frontal Pole
138	LH, Lingual Gyrus
161	LH, Superior Temporal Gyrus, posterior
162	RH, Precuneus Cortex, Cingulate Gyrus, posterior
181	RH, Juxtapositional Lobule Cortex
197	RH, Precentral Gyrus
230	RH, Supramarginal Gyrus, posterior
241	RH, Frontal Pole
242	RH, Frontal Orbital Cortex
258	RH, Lateral Occipital Cortex
278	RH, Frontal Pole
281	RH, Frontal Pole
291	RH, Middle Temporal Gyrus
307	RH, Occipital Pole
308	RH, Lingual Gyrus
317	RH, Frontal Pole
331	RH, Superior Temporal Gyrus, anterior
333	RH, Middle Temporal Gyrus, Superior Temporal Gyrus

### 3.4 Relationship between dMRI and rsfMRI-Networks

We used network statistics to calculate the overlap between differences in dMRI and rsfMRI subnetworks that differed between CHR-P vs. HC groups. Non-parametric permutation testing identified a group of nodes that included left lingual gyrus (node ID 16), right frontal orbital cortex (node ID 242), right lateral occipital cortex (node ID 258) and right superior temporal gyrus (node ID 331).

Assigning the overlapping nodes to known brain networks revealed that the following networks were involved; left and right visual networks, right ventral attention network, and right default mode network.

For each overlapping node, we correlated the pairwise dMRI and rsfMRI connectivity measure with CAARMS severity, SPI-A severity, GAF score, and BACS score.

### 3.5 CAARMS correlations

CAARMS severity significantly correlated with atlas pairs of dMRI connectivity that included left lingual gyrus and right precuneus (r(98) = -0.25, p = 0.03), right lateral occipital cortex and left intracalcarine cortex (r(98) = 0.27, p = 0.02), right posterior parahippocampal gyrus (r(98) = -0.26, p = 0.001) and right anterior inferior temporal gyrus (r(98) = -0.29, p = 0.03), and right superior temporal gyrus and right frontal pole (r(98) = -0.35, p = 0.03).

Finally, there was a single significant correlation for rsfMRI connectivity between right lateral occipital cortex and right precentral / postcentral gyrus (r(98) = -0.34, p = 0.001). This was the only significant correlation with respect to rsfMRI.

### 3.6 SPI-A correlations

For dMRI-connectivity, these significant correlations included right lateral occipital cortex and left precuneus (r(98) = 0.33, p = 0.04), right lingual gyrus (r(98) = -0.28, p = 0.002), right posterior parahippocampal gyrus (r(98) = -0.33, p = 0.009), and right occipital fusiform gyrus (r(98) = -0.3, p = 0.009). There were also significant correlations between right superior temporal gyrus and (r(98) = -0.32, p = 0.005), and (r(98) = -0.27, p = 0.002).

### 3.7 BACS correlations

Significant correlations with BACS total score and dMRI connectivity were found between right frontal orbital cortex and right superior frontal gyrus (r(98) = 0.33, p = 0.02), and between right lateral occipital cortex and right superior parietal lobule (r(98) = -0.31, p = 0.009).

### 3.8 GAF correlations

For GAF score there was a single significant correlation for dMRI between right superior temporal gyrus and right angular gyrus (r(98) = -0.41, p = 0.02).

### 3.9 Relationship between dMRI and rsfMRI, and clinical outcomes in CHR-P participants

Of the CHR-P participants who met APS-criteria, N = 38 CHR-Ps were characterised by persistent APS at 12 months vs. N = 85 who remitted (non-persistent APS). In addition, N = 13 CHR-P participants developed a FEP.

**Fig. 3:**
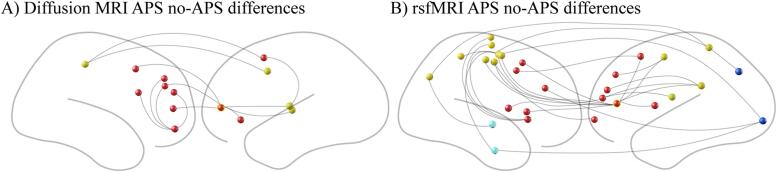
DMRI and rsfMRI differences between persistent APS vs. non-persistent APS.

We identified a single subnetwork from dMRI connectivity differences between persistent APS vs. non-persistent APS groups (Fig. 3A, Table 4) which included 6 left and 8 right hemisphere nodes that included a single hub node left frontal pole, middle frontal gyrus (degree = 6, centrality = 0.65). Of the significant connections between nodes, 19 showed higher connectivity in the APS group and 7 showed higher connectivity in the non-persistent APS group. Effect sizes ranged from between 0.31 and 0.46 with a median of 0.35 (Supplementary M).

**Table 4:** Node ID and Brain Region for dMRI subnetwork 1 for differences between APS and non-persistent APS.

**Node ID**	**Region**
10	LH, Heschl’s Gyrus
40	LH, Precentral Gyrus
49	LH, Superior Frontal Gyrus
84	LH, Insular Cortex
104	LH, Parietal Operculum Cortex, Central Operculum Cortex
110	LH, Frontal Pole, Middle Frontal Gyrus
188	RH, Paracingulate Gyrus
200	RH, Superior Frontal Gyrus
216	RH, Postcentral Gyrus
277	RH, Middle Frontal Gyrus
322	RH, Paracingulate Gyrus, Frontal Pole
323	RH, Frontal Pole
324	RH, Superior Frontal Gyrus
325	RH, Paracingulate Gyrus, Superior Frontal Gyrus

Similarly, we identified two subnetworks that exhibited significant rsfMRI connectivity differences between APS persistent vs. APS non-persistent groups (Fig. 3B, Table 5). The largest subnetwork contained 13 left and 16 right hemisphere nodes. Of the significant connections between nodes, 52 showed higher connectivity in the APS group and 27 showed higher connectivity in the non-persistent APS group. Effect sizes ranged from between 0.31 and 0.45 with a median of 0.36 (Supplementary N).

**Table 5:** Node ID and Brain Region for rsfMRI subnetwork 1 for differences between APS and non-persistent APS.

**Node ID**	**Region**
7	LH, Frontal Pole
25	LH, Paracingulate Gyrus
41	LH, Precentral Gyrus
50	LH, Superior Frontal Gyrus
64	LH, Supramarginal Gyrus, posterior
74	LH, Frontal Pole, Inferior Frontal Gyrus
77	LH, Insular Cortex, Central Opercular Cortex
81	LH, Insular Cortex
94	LH, Lateral Occipital Cortex, superior
115	LH, Frontal Pole
140	LH, Occipital Pole
147	LH, Frontal Pole
156	LH, Middle Frontal Gyrus
183	RH, Paracingulate Gyrus, Cingulate Gyrus, anterior
185	RH, Cingulate Gyrus, anterior
187	RH, Paracingulate Gyrus
197	RH, Precentral Gyrus
198	RH, Precentral Gyrus
203	RH, Precentral Gyrus
205	RH, Precentral Gyrus
206	RH, Precentral Gyrus
210	RH, Superior Parietal Lobule
218	RH, Postcentral Gyrus
247	RH, Insular Cortex
248	RH, Insular Cortex
259	RH, Supramarginal Gyrus, posterior
274	RH, Central Opercular Cortex
305	RH, Temporal Fusiform Cortex, posterior
332	RH, Superior Temporal Gyrus

Finally, we examined the overlap between dMRI and rsfMRI connectivity differences between persistent APS vs. non-persistent APS groups and observed several nodes. These included left Heschl’s gyrus (p < 0.02), left precentral gyrus (p < 0.03), left frontal pole (p < 0.03), left insular cortex (p < 0.02), left posterior parahippocampal gyrus (p < 0.02), right posterior supramarginal gyrus (p < 0.03) and right frontal pole (p < 0.01). These nodes conform to the left auditory network, left cingulo-opercular network, left frontoparietal network, left cingulo-opercular network, left visual network, right auditory network and the right frontoparietal network.

## 4 Discussion

We compared dMRI and rsfMRI-informed connectivity patterns between CHR-P individuals and HC to determine whether large-scale networks in CHR-Ps involve a dysconnectivity syndrome that involves overlapping structural and functional deficits. Our findings highlight that both dMRI and rsfMRI networks are characterised by both hyper- and hypo-connectivity. Importantly, we observed significant overlap between dMRI and rsfMRI-data in frontal, temporal and occipital regions, suggesting that dMRI and rsfMRI connectome anomalies may arise from common disturbances in underlying neural circuitry. Moreover, abnormalities in dMRI and rsfMRI networks correlated with the severity of CHR-Ps symptoms, cognitive deficits, and clinical outcomes.

Our study contributes to a growing body of evidence indicating alterations in large-scale dMRI and rsfMRI networks in CHR-Ps (Collin et al., 2017, Nelson et al., 2017, Skudlarski et al., 2010). Specifically, we identified subnetworks in both dMRI- and rsfMRI-networks that spanned frontal, occipital, parietal, and temporal regions across hemispheres that differed between CHR-P and HC, suggesting a distributed impairment across cortical regions. Importantly, these subnetworks involved several hub nodes highlighting that connectivity disturbances in CHR-P converge on core regions that are important for network organisation (Wang et al., 2017).

An important finding of our analysis is the identification of common regions of dMRI and rsfMRI connectivity anomalies in CHR-Ps. We identified areas that were shared between dMRI and rsfMRI-defined networks that differed between CHR-Ps and HC which included left lingual gyrus, right frontal orbital cortex, right lateral occipital cortex, and right superior temporal gyrus. Furthermore, these brain regions could be assigned to networks, such as the left and right visual networks, right ventral attention network, and right default mode network. These data are consistent with previous findings that have shown functional and structural network abnormalities in CHR-P populations (Sasabayashi et al., 2023, Schwarzer et al., 2022, Cowan et al., 2022, Haas et al., 2020) as well with extensive evidence on the presence of sensory and cognitive deficits (Oeztuerk et al., 2021, Damme et al., 2023).

Correlational analyses indicated significant relationships between the severity of CHR-P symptoms, cognitive deficits, and impaired functioning with dMRI, and only modestly with rsfMRI connectivity measures, which involved patterns of both hyper- and hypoconnectivity. Accordingly, these data highlight that the symptomatic and cognitive impairments in CHR-Ps may result from a complex pattern of aberrant network dynamics as opposed to localised abnormalities in circumscribed brain regions (Uhlhaas, 2015).

Finally, CHR-P individuals with persistent APS were characterised by aberrant dMRI and rsfMRI connectivity compared to non-persistent APS. Specifically, we observed that frontal and parietal nodes mediated differences between the two groups. Moreover, there was an overlap between dMRI and rsfMRI connectivity in auditory, cingulo-opercular, frontoparietal and visual networks, which has previously been involved in dMRI and rsfMRI abnormalities in schizophrenia (Alonso-Solís et al., 2015, Zemánková et al., 2018, Jiang et al., 2020). Finally, our data align with previous findings demonstrating functional dysconnectivity in resting-state fMRI data that predicted clinical outcomes in CHR-Ps (Anticevic et al., 2015, Fabro et al., 2021). However, a recent study investigating WM differences in CHR-P individuals did not observe relationships with clinical outcomes (Biase et al., 2021).

The overlap between dMRI and rsfMRI-informed connectivity anomalies raises the question regarding the underlying mechanisms. Abnormalities in rsfMRI networks have been linked to aberrant glutamatergic neurotransmission that could potentially lead to complex patterns of aberrant functional connectivity in CHR-Ps and early-stage psychosis (Anticevic et al., 2015, Krystal and Anticevic, 2015). In contrast, impairments in WM pathways have been linked to aberrant brain development (Karlsgodt, 2020, Yu et al., 2022) and specifically to the role of oligodendrocites (Yu et al., 2022). Interestingly, oligodendrocytes also express functional N-Methyl-d-Aspartate (NMDA) receptors (Paoletti and Neyton, 2007). Accordingly, one possibility is that a disturbance in the balance between excitation and inhibition (E/I-balance) underlies both functional and anatomical dysconnectivity anomalies in psychosis.

We would like to note several limitations of the current study. Firstly, we used a brain atlas based on group rsfMRI, which may limit the generalisability of our results to previous studies. Future research should therefore converge on an optimal parcellation to improve the generalisability and replicability of network-based analysis. Moreover, the atlas used in the current study only included cortical regions. Finally, our study sample was not sufficiently large to assess the relationship between dMRI and rsfMRI network parameters and the transition to psychosis in CHR-Ps. Clinical outcomes in CHR-Ps can include transitions to schizophrenia but also affective psychosis (Yoviene Sykes et al., 2020) as well as other psychotic disorders, such psychosis not otherwise specified.

In conclusion, our study revealed robust albeit circumscribed differences in both dMRI and rsfMRI connectivity in CHR-P individuals, suggesting that overlapping disturbances in large-scale dMRI and rsfMRI networks may be present in CHR-P individuals. In addition, these large-scale network alterations correlated with both symptoms and cognitive impairments as well as with clinical outcomes in CHR-Ps, highlighting that both dMRI and rsfMRI connectivity disturbances are likely to play an important role in the pathophysiology of emerging psychosis.

## CRediT authorship contribution statement

**Angus Paton:** Writing – review & editing, Writing – original draft, Visualization, Validation, Software, Resources, Project administration, Methodology, Investigation, Formal analysis, Data curation, Conceptualization. **Tineke Grent- ’t-Jong:** Data curation. **Ruchika Gajwani:** Writing – review & editing. **Joachim Gross:** Writing – review & editing. **Andrew I. Gumley:** Writing – review & editing. **Stephen M. Lawrie:** Writing – review & editing. **Matthias Schwannauer:** Writing – review & editing. **Frauke Schultze-Lutter:** Writing – review & editing. **Peter J. Uhlhaas:** Writing – review & editing, Writing – original draft, Visualization, Validation, Supervision, Resources, Project administration, Methodology, Investigation, Funding acquisition, Formal analysis, Data curation, Conceptualization.

## Funding

The study was supported by the Medical Research Council (MR/L011689/1).

## Declaration of Competing Interest

The authors declare that they have no known competing financial interests or personal relationships that could have appeared to influence the work reported in this paper.

## Data Availability

Data will be made available on request.
