# Supplementary material for: Overlapping structural and functional connectivity disruptions in clinical high-risk for psychosis participants: A network analysis study
Source: Neuroimage Clin. 2025 May 12;47:103803. doi: 10.1016/j.nicl.2025.103803 (PMC12173700; doi:10.1016/j.nicl.2025.103803)

Supplementary A: Comparing standard deviations for removing subjects due to motion deviation from the group mean. For diffusion MRI we calculate mean relative RMS and for rsfMRI we calculate average framewise displacement across all 6 motion parameters. We then calculate the deviation from the mean for each subject. Deviation for each subject is plotted, along with reference lines for one standard deviation (blue), 2 standard deviations (magenta) and 3 standard deviations (red).


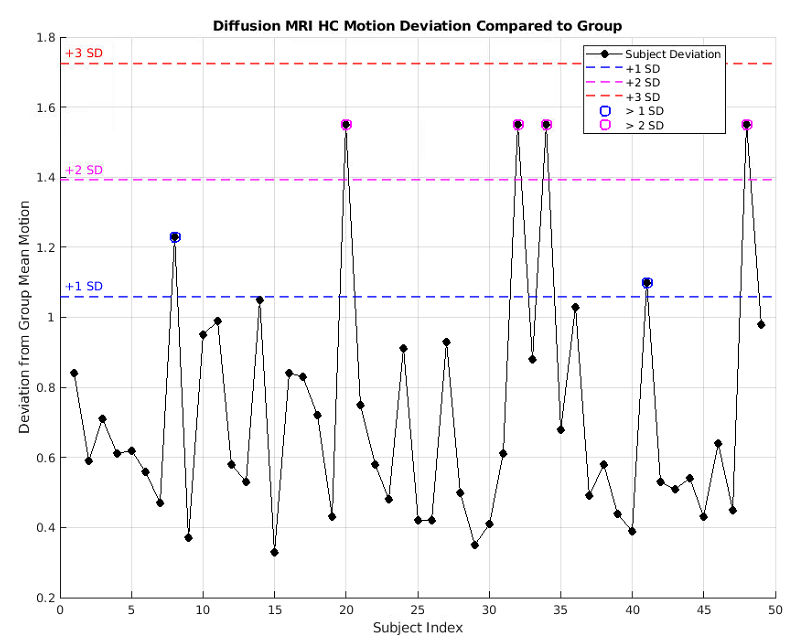


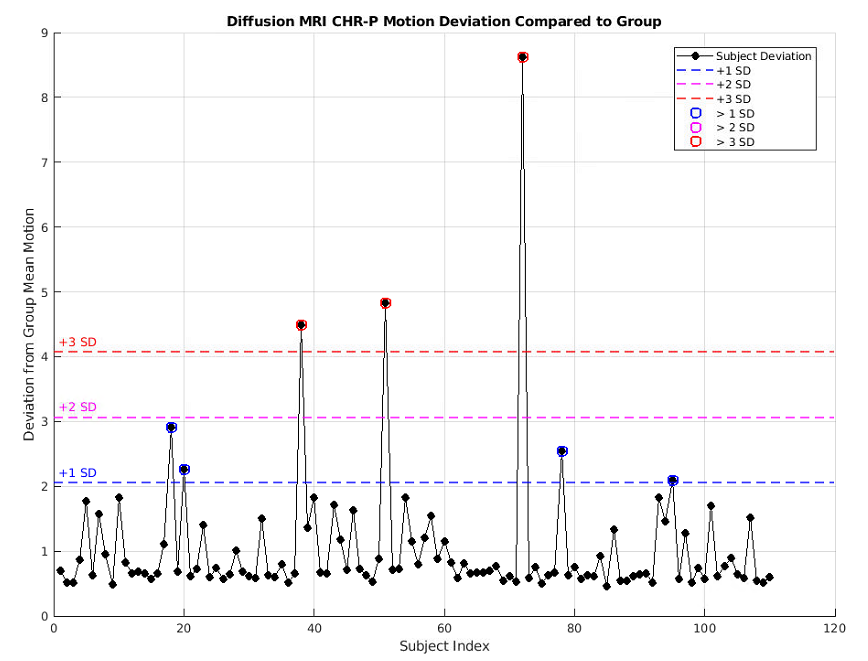


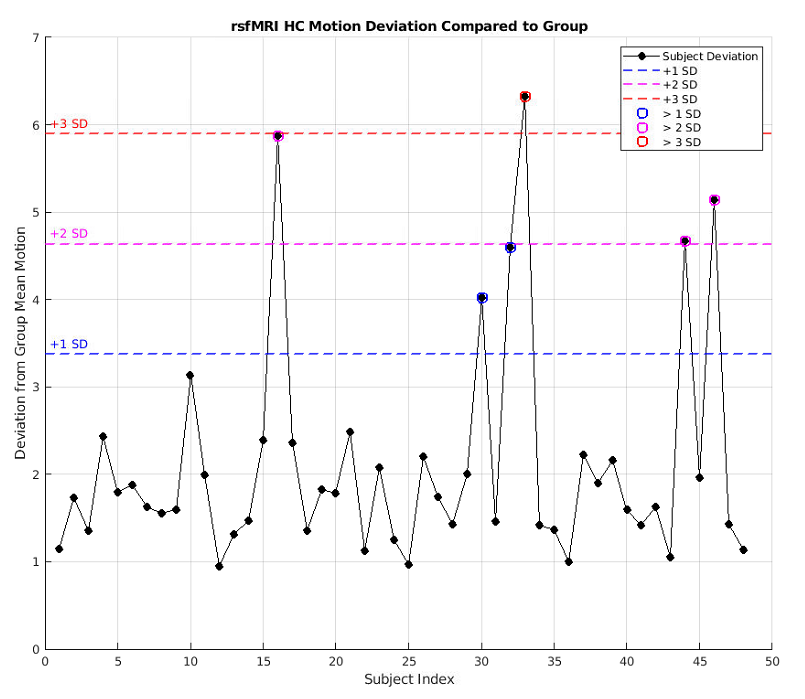


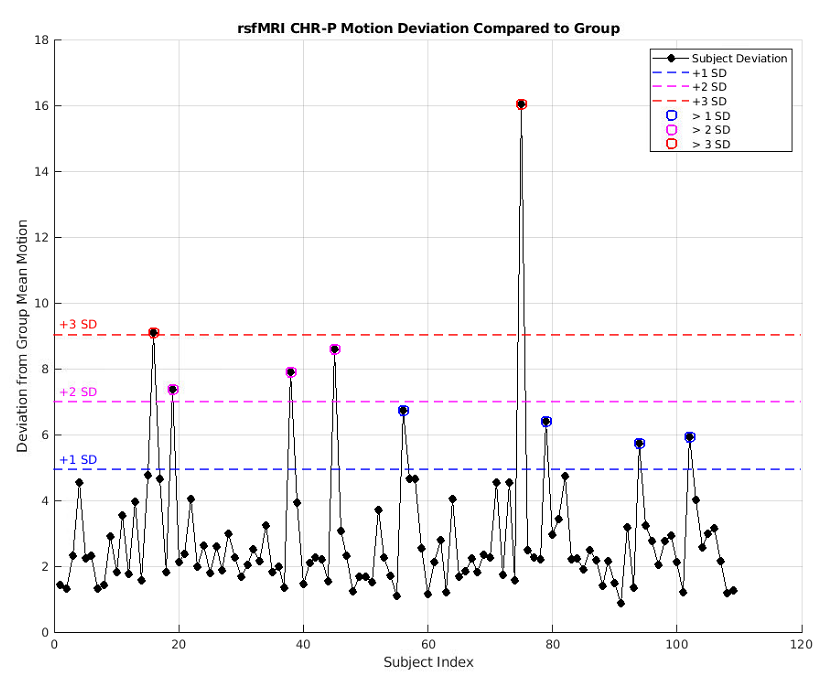


Supplementary B: Absolute and relative motion parameters for the whole sample for diffusion tensor imaging data


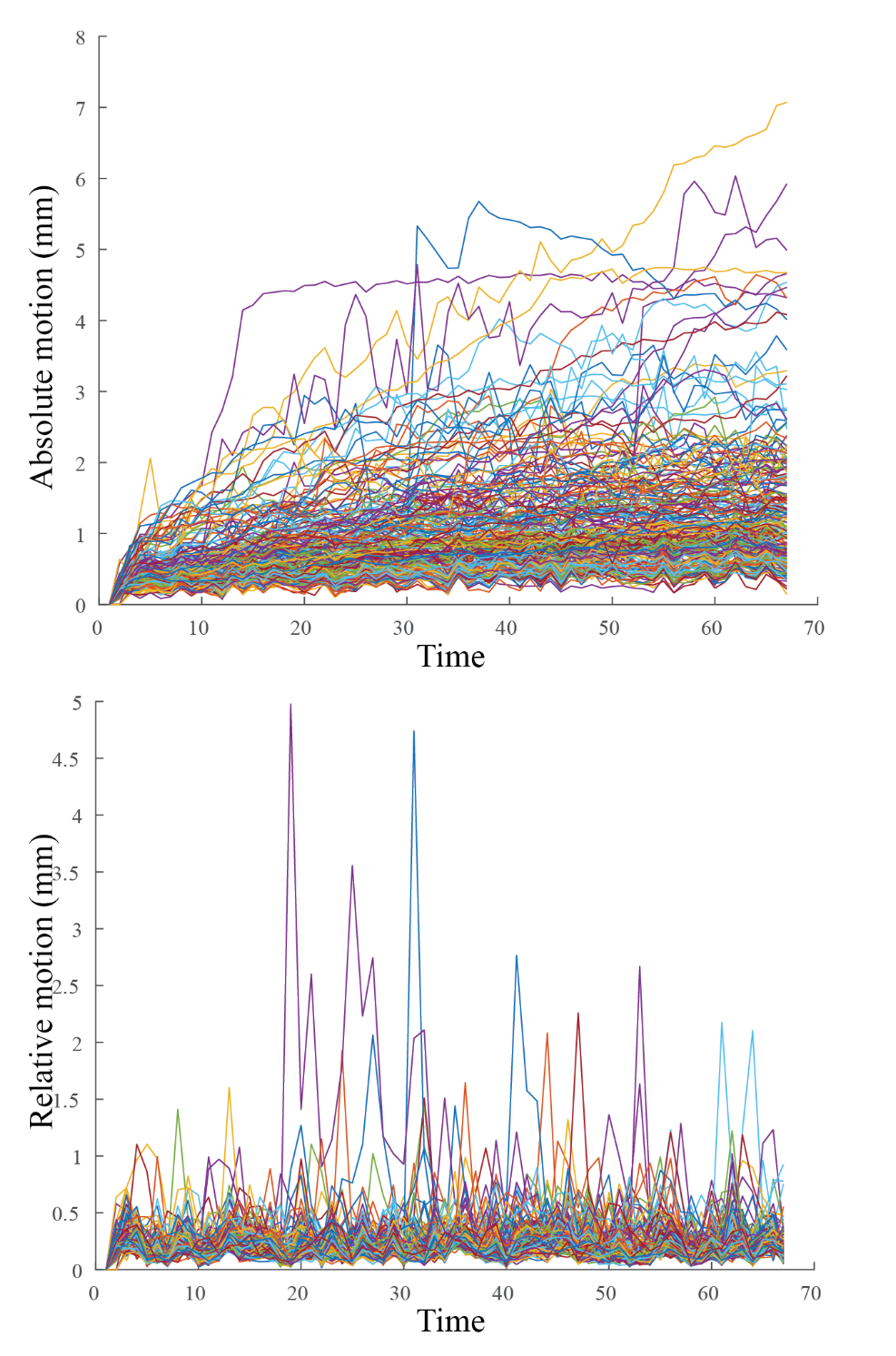


Supplementary C: Resting state motion correction summary statistics. Mean and maximum motion correction displacement for the CHR-P and control groups for resting state data.


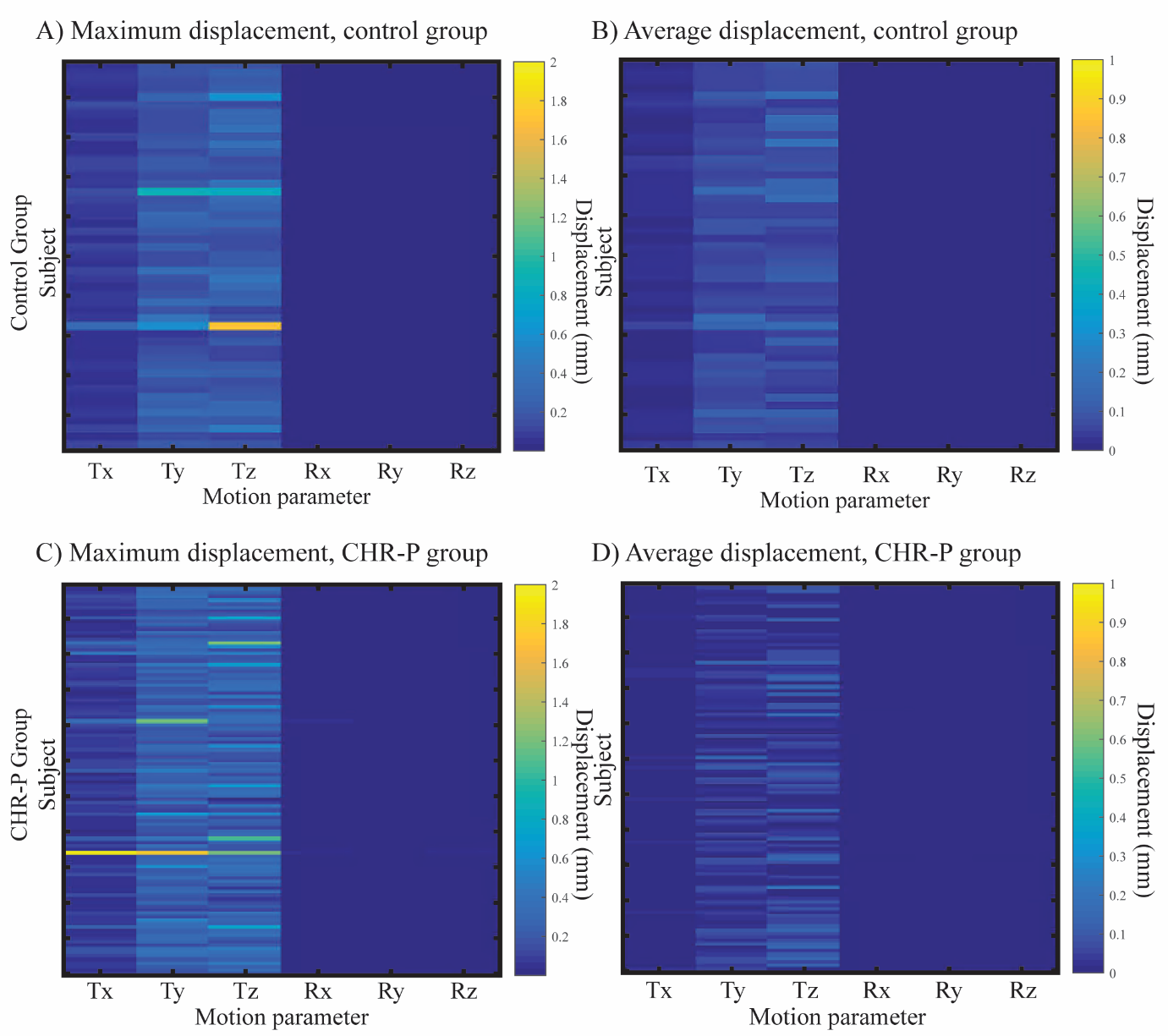


Supplementary D: Summary of Parcel Labels from the Gordon 2014 Atlas


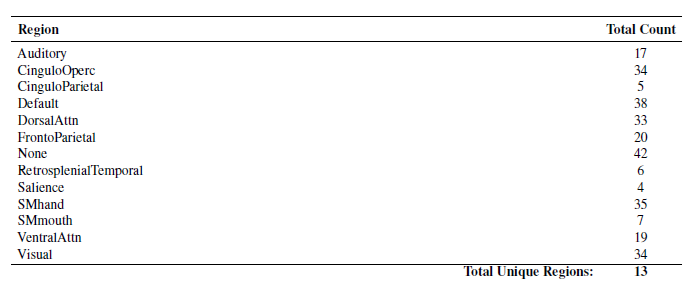


Supplementary E: Summary of Parcel Relabelling Using the Harvard Oxford Atlas as Reference


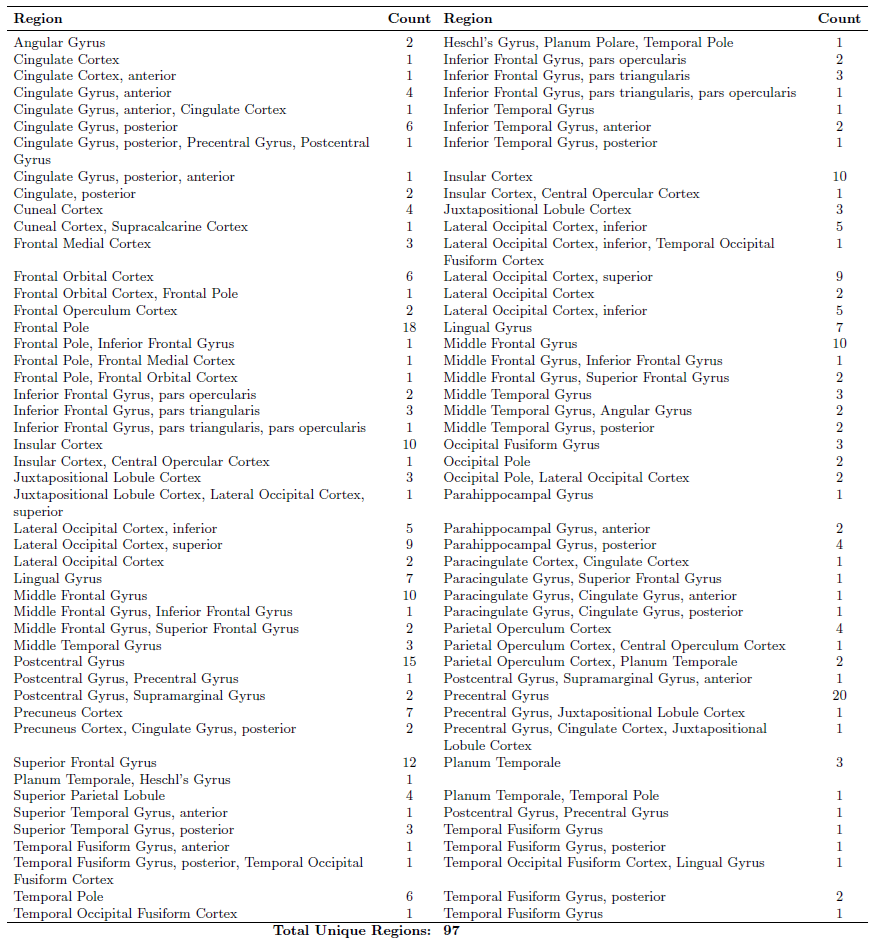


Supplementary F: Node ID and Brain Region for dMRI subnetwork 2 for differences between CHR-P and HC. Edge weights are reporting directionality of effect size.


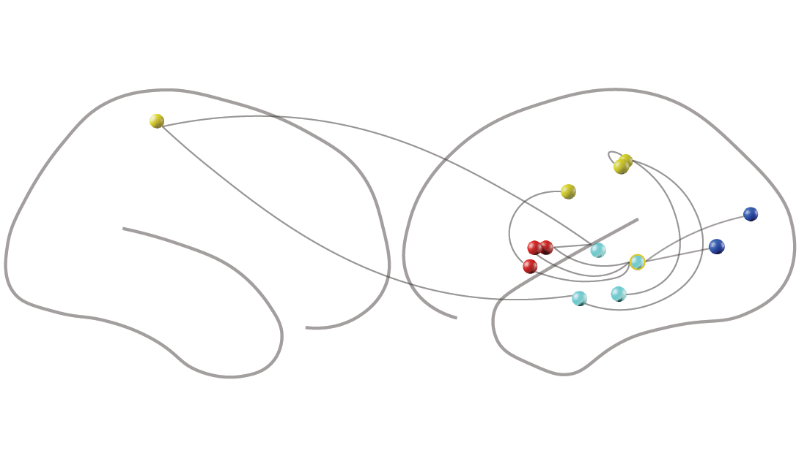


| **Node ID** | **Region** |
| --- | --- |
| 21 | LH, Precuneus Cortex, Precentral Gyrus, Cingulate Cortex |
| 53 | LH, Postcentral Gyrus |
| 55 | LH, Supramarginal Gyrus, anterior |
| 62 | LH, Supramarginal Gyrus, posterior |
| 66 | LH, Heschl’s Gyrus |
| 76 | LH, Frontal Operculum Cortex |
| 81 | LH, Insular Cortex |
| 82 | LH, Insular Cortex |
| 99 | LH, Lateral Occipital Cortex, inferior |
| 126 | LH, Middle Temporal Gyrus, temporooccipital |
| 127 | LH, Middle Temporal Gyrus |
| 137 | LH, Occipital Pole, Cuneal Cortex |
| 214 | RH, Postcentral Gyrus |


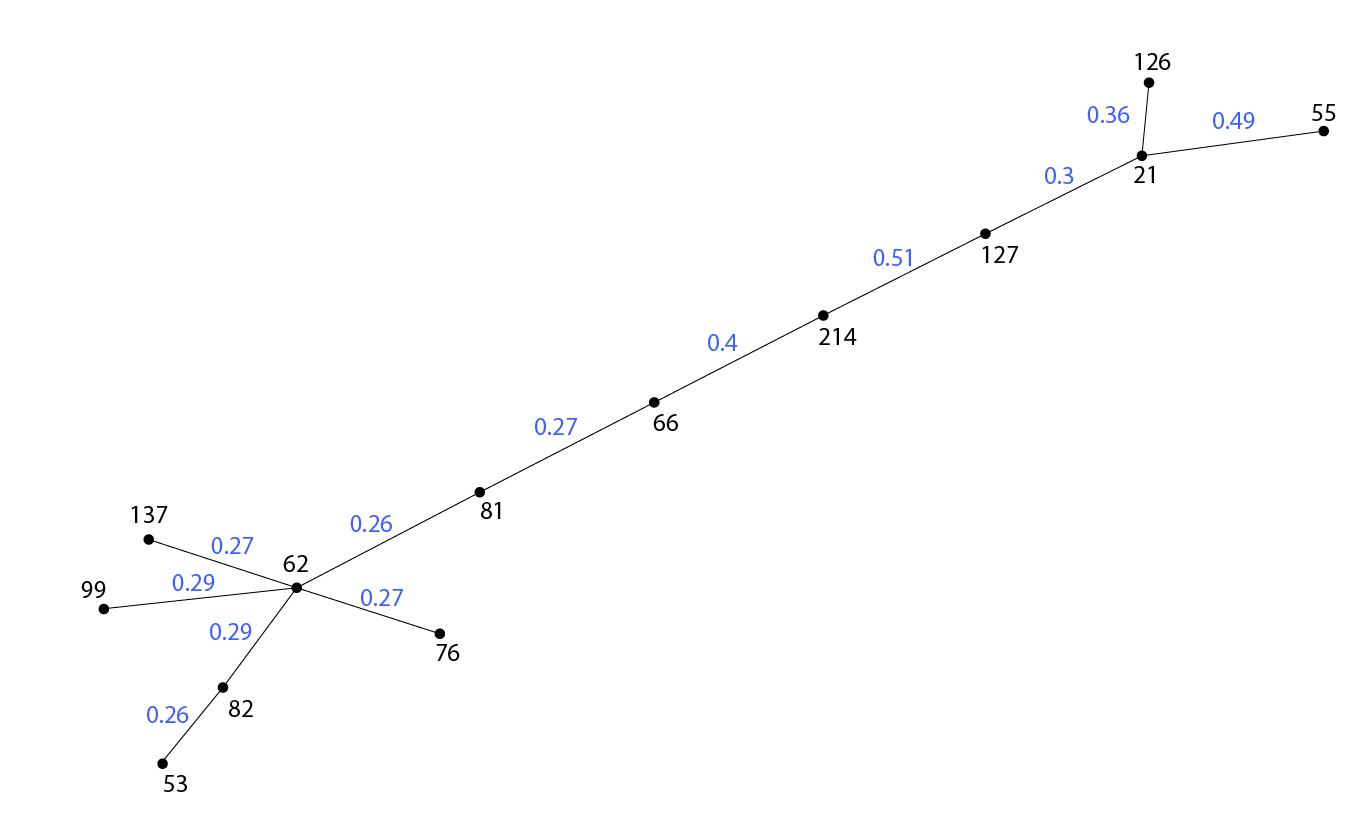


Supplementary H: Node ID and Brain Region for rsfMRI subnetwork 2 for differences between CHR-P and HC. Edge weights are reporting directionality of effect size.


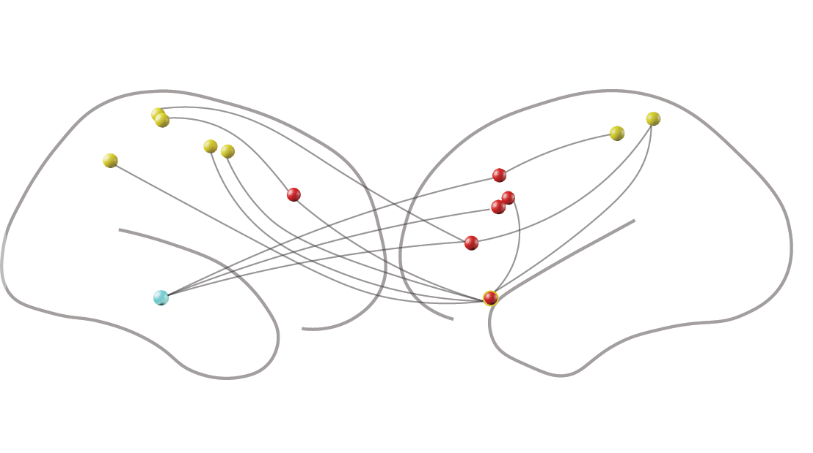


| **Node ID** | **Region** |
| --- | --- |
| 28 | LH, Cingulate Gyrus, anterior |
| 35 | LH, Postcentral Gyrus |
| 38 | LH, Postcentral Gyrus |
| 74 | LH, Frontal Pole, Inferior Frontal Gyrus |
| 109 | LH, Inferior Frontal Gyrus, pars triangularis |
| 120 | LH, Frontal Orbital Cortex |
| 154 | LH, Superior Frontal Gyrus |
| 188 | RH, Paracingulate Gyrus |
| 193 | RH, Postcentral Gyrus |
| 194 | RH, Precentral Gyrus |
| 195 | RH, Precentral Gyrus, Cingulate Cortex, Juxtapositional Lobulae Cortex |
| 214 | RH, Postcentral Gyrus |
| 261 | RH, Angular Gyrus, Superior Parietal Lobule |
| 289 | RH, Middle Temporal Gyrus, posterior |


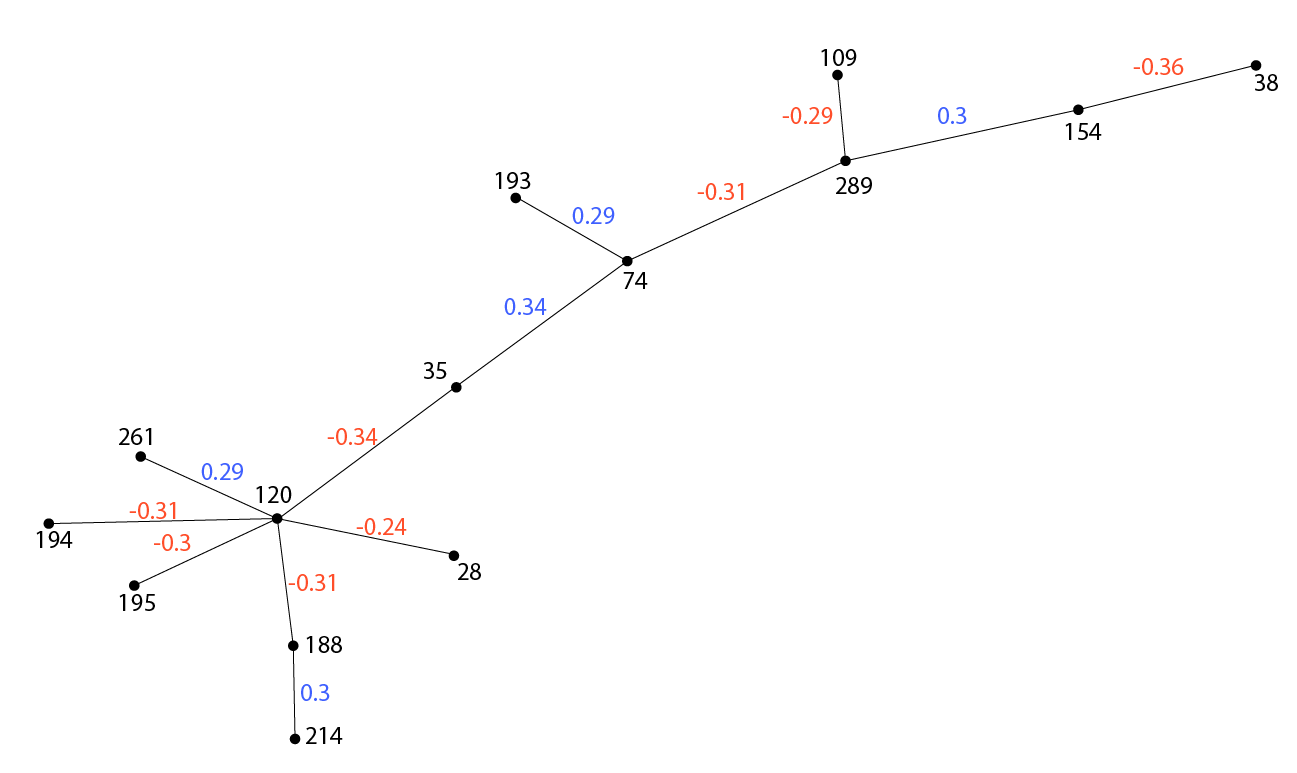


Supplementary I: Node ID and Brain Region for rsfMRI subnetwork 3 for differences between CHR-P and HC. Edge weights are reporting directionality of effect size.


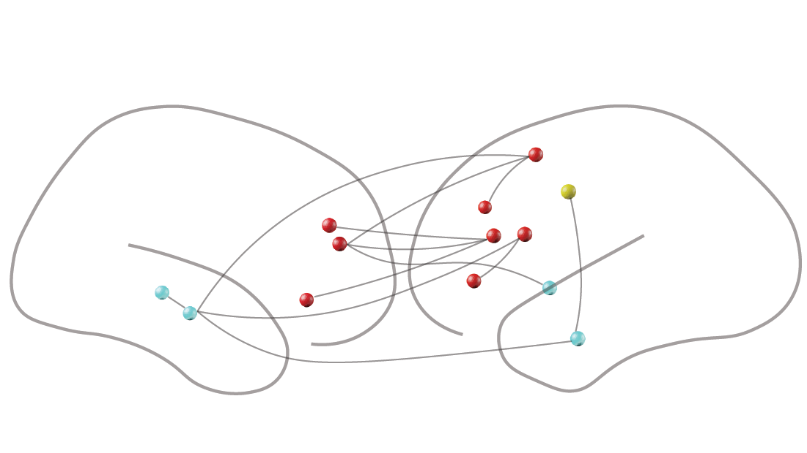


| **Node ID** | **Region** |
| --- | --- |
| 29 | LH, Paracingulate Gyrus |
| 53 | LH, Postcentral Gyrus |
| 72 | LH, Insular Cortex |
| 75 | LH, Inferior Frontal Gyrus, pars triangularis |
| 85 | LH, Frontal Orbital Cortex |
| 112 | LH, Inferior Frontal Gyrus, pars opercularis |
| 128 | LH, Inferior Temporal Gyrus, anterior |
| 158 | LH, Middle Frontal Gyrus |
| 168 | RH, Frontal Pole |
| 236 | RH, Frontal Pole |
| 283 | RH, Frontal Orbital Cortex, Frontal Pole |
| 296 | RH, Parahippocampal Gyrus, anterior |
| 313 | RH, Parahippocampal Gyrus, posterior, Lingual Gyrus |


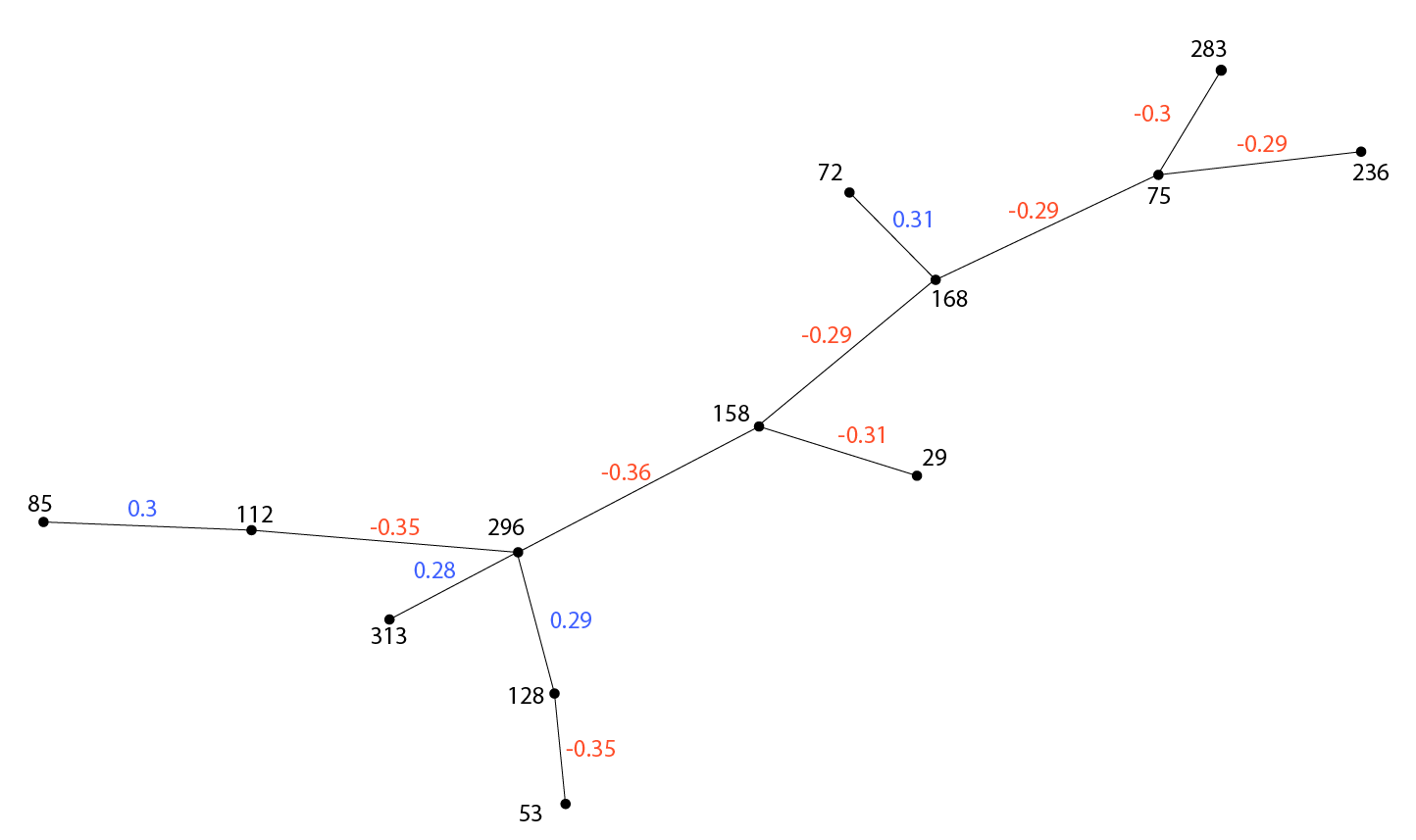


Supplementary J: Node ID and Brain Region for rsfMRI subnetwork 2 for differences between APS and non-Persistent APS. Edge weights are reporting directionality of effect size.


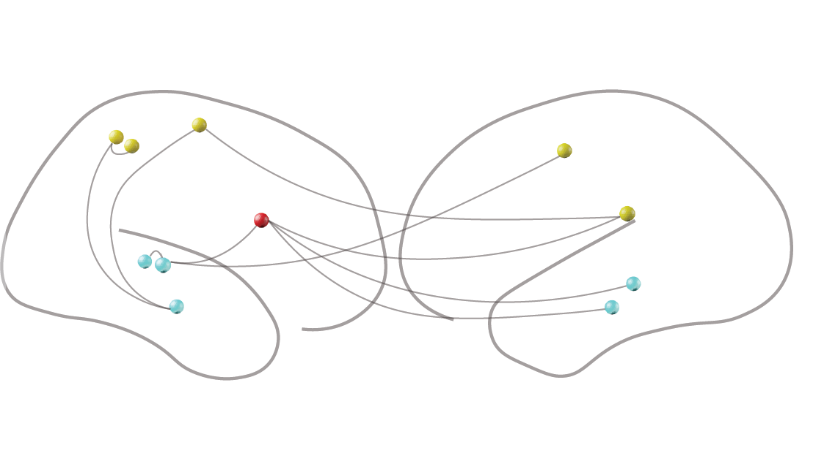


| **Node ID** | **Region** |
| --- | --- |
| 18 | LH, Parahippocampal Gyrus, posterior, Temporal Fusiform Cortex |
| 40 | LH, Precentral Gyrus |
| 103 | LH, Parietal Operculum Cortex |
| 144 | LH, Parahippocampal Gyrus, posterior |
| 176 | RH, Lingual Gyrus |
| 178 | RH, Cingulate, posterior |
| 201 | RH, Precentral Gyrus |
| 231 | RH, Supramarginal Gyrus, posterior |
| 251 | RH, Superior Parietal Lobule |
| 262 | RH, Superior Parietal Lobule |
| 275 | RH, Inferior Frontal Gyrus, pars opercularis |
| 295 | RH, Parahippocampal Gyrus, posterior |


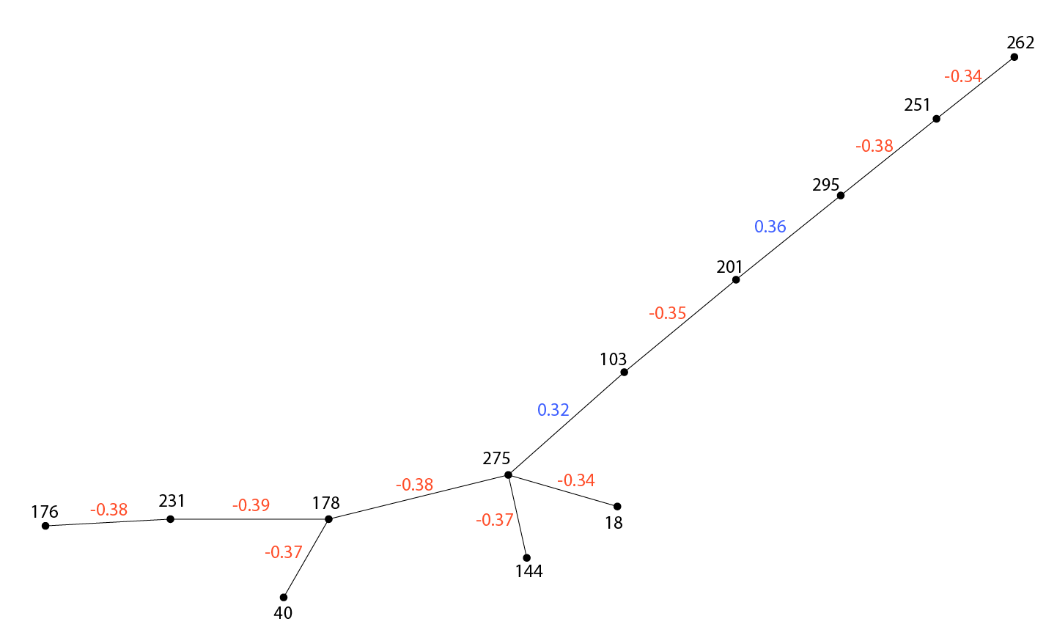


Supplementary K: Effect size and directionality reported as edge weights for dMRI CHR-P versus HC. Nodes are denoted in black, positive directionality in blue and negative directionality in red.


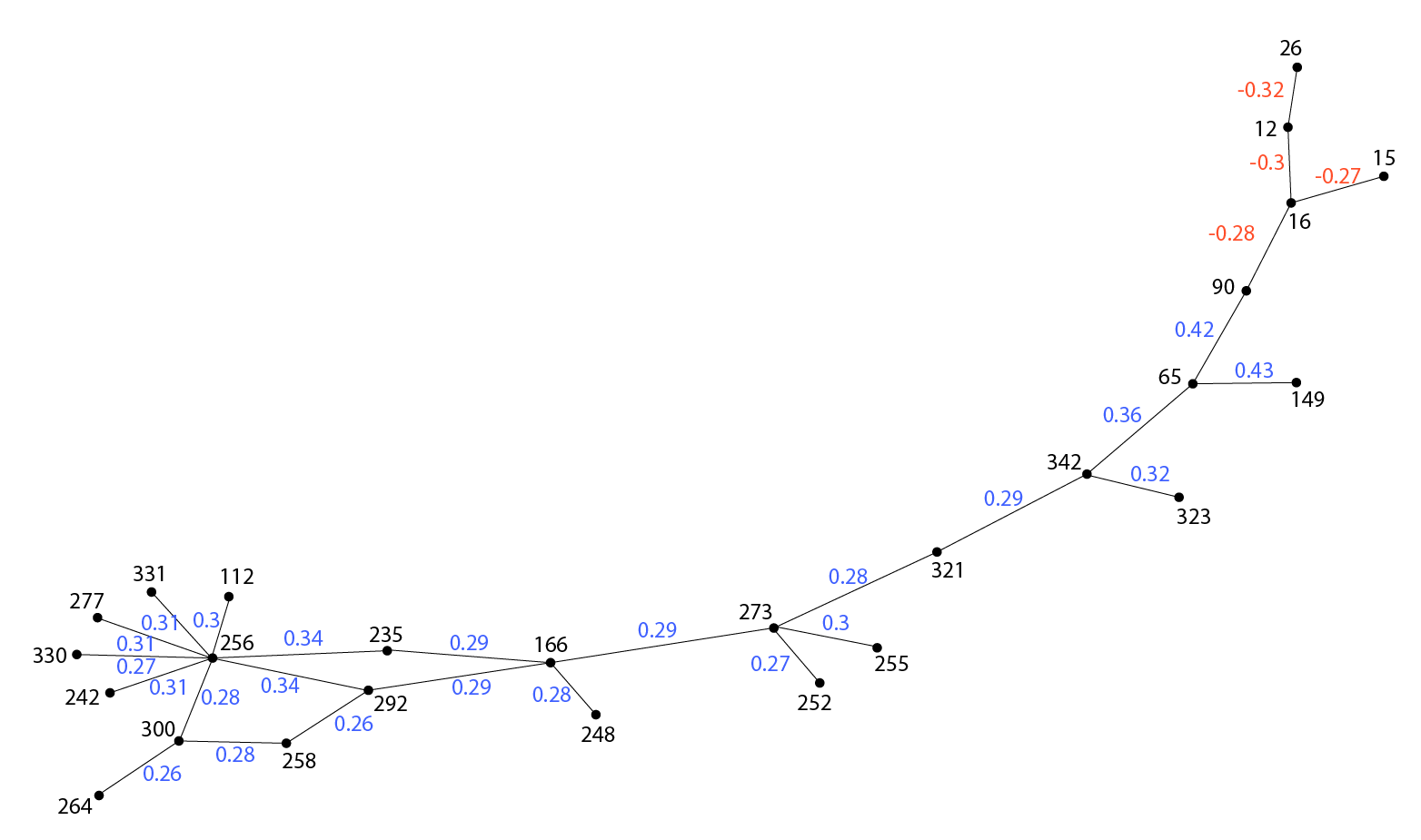


Supplementary L: Effect size and directionality reported as edge weights for rsfMRI CHR-P versus HC. Nodes are denoted in black, positive directionality in blue and negative directionality in red.


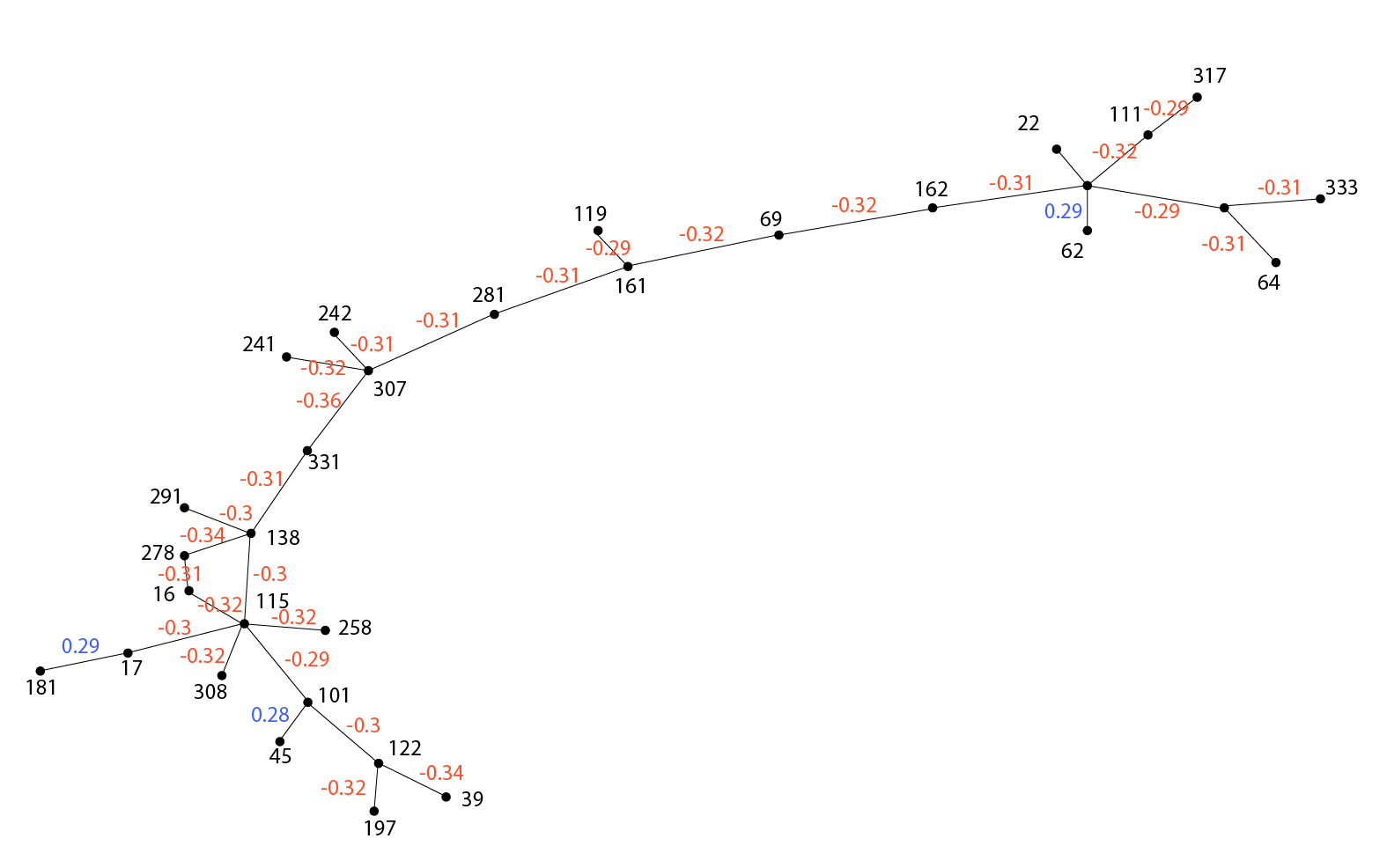


Supplementary M: Effect size and directionality reported as edge weights for dMRI APS versus non-persistent APS. Nodes are denoted in black, positive directionality in blue and negative directionality in red.


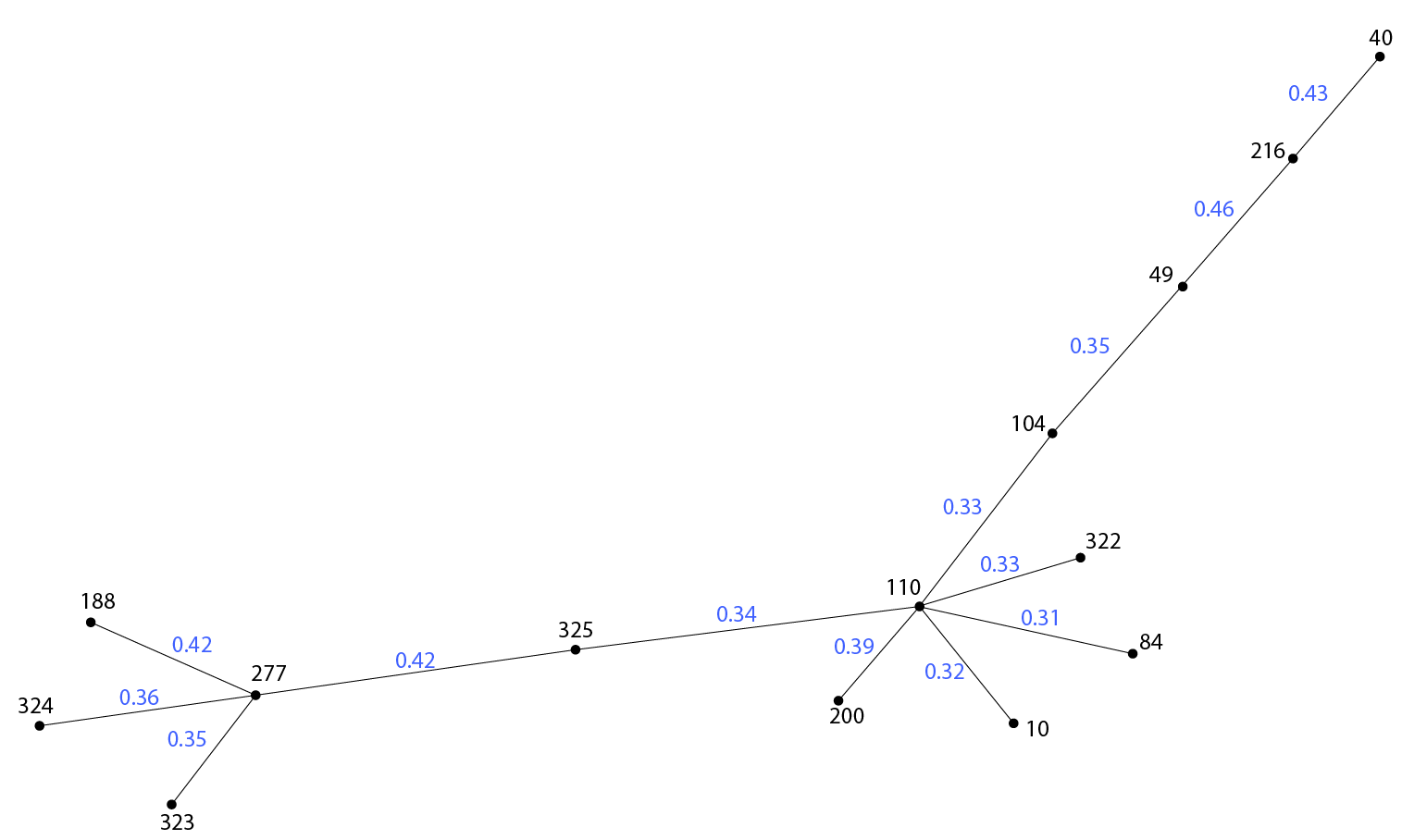


Supplementary N: Effect size and directionality reported as edge weights for dMRI APS versus non-persistent APS. Nodes are denoted in black, positive directionality in blue and negative directionality in red.


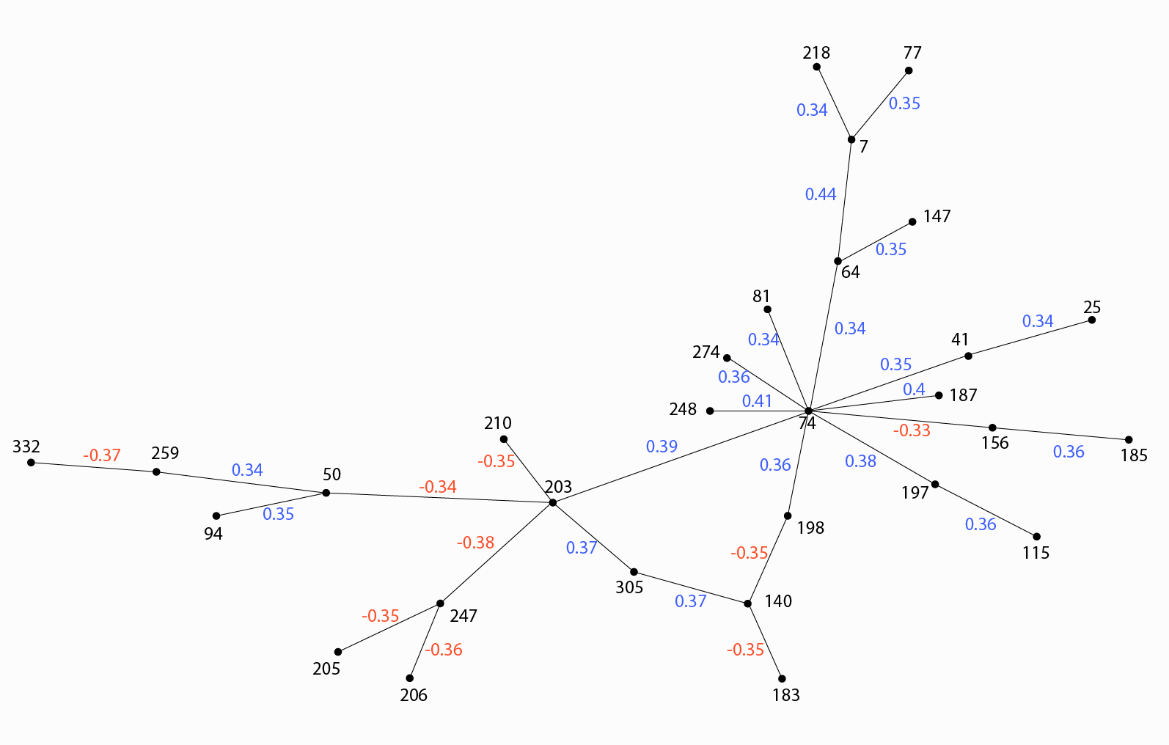

Supplement: Supplementary Data 1 [file mmc1.docx]
